# Investigation of the Properties of Polyethylene and Ethylene-Vinyl Acetate Copolymer Blends for 3D Printing Applications

**DOI:** 10.3390/polym15204129

**Published:** 2023-10-18

**Authors:** Azamat Slonov, Ismel Musov, Azamat Zhansitov, Azamat Khashirov, Aslanbek Tlupov, Khasan Musov, Elena Rzhevskaya, Irina Fomicheva, Andrey Potapov, Svetlana Khashirova

**Affiliations:** 1 Laboratory of Technology of Polymer Materials and Composites, Tula State University, Prospekt Lenina 92, 300012 Tula, Russia; 2 Progressive Materials and Additive Technologies Center, Kabardino-Balkarian State University Named after H.M. Berbekov, St. Chernyshevsky, 173, 360004 Nalchik, Russia

**Keywords:** fused deposition modeling (FDM), polyethylene, polypropylene, ethylene-vinyl acetate copolymer, polymer blends

## Abstract

3D printing of polyolefins, such as polyethylene (PE) and polypropylene (PP), is of great practical interest due to the combination of high properties of these materials. However, the use of these materials in 3D printing is associated with many problems due to their high rate of crystallization, which causes shrinkage and warpage of the printed object. In this regard, blends of PE and ethylene-vinyl acetate copolymer (EVA) of various compositions were investigated for 3D printing. It was found that with an increase in the concentration of EVA, an increase in the pseudoplastic effect and amorphization of PE occurs. It has been shown that with an increase in the EVA content, the degree of crystallinity of PE decreases slightly (by 11% at a content of 80% EVA); however, a significant decrease in the rate of crystallization of PE is observed (by 87.5% at the same EVA concentration). It was found that PE and EVA are completely compatible in the amorphous phase and partially compatible in the crystalline phase, which leads to a slight decrease in the melting point of PE. The introduction of EVA also leads to a significant increase in impact strength: the maximum value is achieved at a 50/50 ratio, which is five times the value of the initial PE and two times the value of the initial EVA. At the same time, it was revealed that EVA leads to a gradual decrease in the elastic modulus and strength of PE, the change of which generally obeys the additivity rule. The resulting printing filaments are characterized by a certain ovality due to their shrinkage, which decreases with increasing EVA content and reaches a minimum value at a PE/EVA ratio of 30/70. This composition also demonstrates the lowest shrinkage of the printed sample and higher processability during printing.

## 1. Introduction

3D printing or additive manufacturing is a new technology that allows the creation of three-dimensional objects based on a digital model using layer-by-layer deposition of material [1]. Today, many 3D printing methods are known, which differ in the way of layers deposition and the nature of materials (metals, ceramics, polymers) [2]. In the additive manufacturing of polymers, the most popular technologies are selective laser sintering (SLS), which uses a polymer powder; fused deposition modeling (FDM), which uses thermoplastic filaments; and stereolithography (SLA), where an oligomeric resin serves as a raw material.

FDM printing has become the most widespread among polymer additive manufacturing technologies [3]. In this method, the object is formed by the deposition of a polymer melt, which is fused with the previous layer. This extrusion printing method was developed by Stratasys in the late 1980s. Until 2012, the range of materials for FDM 3D printing, especially for more affordable printer models, was limited to polylactide (PLA) and acrylonitrile butadiene styrene (ABS), and they still remain in the most demand in this technology. However, many higher-grade thermoplastics are now commercially available, such as polycarbonate, polysulfones, polyamides, polyethylene terephthalate, as well as polyimides and polyketones [4]. All of these materials have their advantages and disadvantages, and therefore, the expansion of the range of materials for FDM printing remains relevant.

There are a number of requirements for the successful use of materials in 3D printing using the FDM method [5]:

- Firstly, the material must exit the die of a certain diameter with a given volumetric velocity. Obviously, this parameter depends on the melt viscosity (*η*) at a given printing temperature (T_p_) and shear rate (*γ*);
- Secondly, it is necessary that after laying on the working table, the material maintains a certain height (thickness) and geometry (semi-rectangular), which depends on the surface energy (*γ*_se_) and melt density (ρ);
- Thirdly, the laid layer should serve as a reliable support for the subsequent layer, which creates a certain pressure on it. The degree of deformation of the laid layer from the subsequent one (preferably < 10%) depends on the viscoelastic properties of the material, i.e., on the ratio of loss modulus (E″) and accumulation modulus (E′) under printing conditions. In addition, in order to realize the possibility of obtaining objects with complex geometry and varying degrees of filling, the material must provide overlapping of the rasters without sagging and also not deform under the force of the flow when laying the next layer, especially when the flow is reoriented, which also depends on the viscoelastic properties;
- Fourthly, the integrity and accuracy of the geometry of the entire structure must be maintained. Different shrinkage of the layers can cause a violation of the geometry of the entire construction object, or residual stresses can find a way out through the formation of a local crack.

The last condition, i.e., shrinkage and warping of parts during printing, is one of the main reasons that hinders the widespread industrial use of FDM printing and limits the type of polymer matrices used. In particular, this problem prevents the use of materials such as polyethylene (PE) and polypropylene (PP), which have an excellent combination of properties and are affordable. Significant shrinkage and warping due to the high degree of crystallinity and the rate of crystallization of these materials make it very difficult to print parts with precise geometry from them [6].

It is noted that one of the initial problems of using PP in printing is poor adhesion of the first layer to the desktop, which is associated with the absence of functional groups on the surface, low polarity, and surface energy [7,8]. A study of the influence of printing temperature and layer thickness on the impact strength of PP revealed that at a higher printing temperature and low thickness, the highest properties are provided, which is associated with good raster connection and the formation of β-crystallites [9,10]. Additionally, the formation of β-crystallites was detected at elevated chamber temperatures [11].

Many authors have explored the possibility of reducing shrinkage and warpage by introducing various fillers, in particular glass [7] or carbon fibers [12], as well as mixing with other thermoplastics [13].

Research on the possibility of using PE in 3D printing is quite limited. It is reported that increasing the nozzle temperature to 260 °C leads to a decrease in warpage, which the authors characterize using the deviation of the sample from the plane [14]. The authors associate this effect with a change in the structure of PE, which is initiated by the increased temperature of the printer extruder head. There are also works that study composite materials for 3D printing based on PE with titanium oxide [15] and aluminum oxide [16,17]. A study of composites based on high-density polyethylene (HDPE) with graphene and low-density polyethylene (LDPE) showed that the introduction of a polymer with low crystallinity leads to a decrease in the crystallization temperature and the degree of crystallinity of HDPE [18]. Commercially available materials for printing based on PP are also polymer mixtures with reduced crystallinity [4].

Based on this, in this work, the effect of an ethylene-vinyl acetate (EVA) copolymer on the properties of HDPE was investigated in order to identify the possibility of using these mixtures for 3D printing using the FDM method.

## 2. Materials and Methods

The objects of study were HDPE grade PE2NT22-12 (KazanOrgSintez, Kazan, Russia) with a melt flow rate (MFR) of 6.6 g/10 min, an ethylene-vinyl acetate copolymer (EVA) grade 11306-075, with a vinyl acetate content of 12% (KazanOrgSintez, Kazan, Russia). Blends of PE and EVA (PE/EVA) were prepared in the following ratios: 100/0; 80/20; 70/30; 60/40; 50/50; 40/60; 30/70; 20/80; 0/100.

To obtain polymer-polymer composites, a PJSZ twin-screw microextruder from Haitai Machinery (Ningbo, China) with L/D = 30 was used. The extrusion was carried out at a maximum temperature of 200 °C. To obtain samples for testing, an injection molding machine SZS-20 of the same company was used. Injection molding took place at a cylinder temperature of 235 °C and a mold temperature of 60 °C.

Dog-bone-shaped specimens with dimensions in accordance with GOST 112 62-80 were used for mechanical tensile tests. The tests were carried out on a CT-TCS 2000 machine from Gotech Testing Machine (Taichung, Taiwan) at a temperature of 23 °C. At least five samples were tested. To determine theimpact strength, samples with dimensions of 80 × 10 × 4 mm^3^ were tested with notch according to the Izod method in accordance with GOST 19109-84 on GT-7045-MD testing equipment from Gotech Testing Machine (Taichung, Taiwan). At least 10 samples were tested.

The MFR was determined at a temperature of 190 °C and a load of 2.16 kgf on a PTR-LAB-02 instrument from LOIP (St. Petersburg, Russia). The melt viscosity was determined on a Dynisco LCR 7001 capillary rheometer (Franklin, MA, USA) at a temperature of 190 °C using a capillary with a diameter of 0.75 mm and L/D = 16 and 0.5 mm and L/D = 3.

The melting and crystallization temperatures were determined using differential scanning calorimetry (DSC) on a DSC 4000 device from PerkinElmer (Waltham, MA, USA) and dynamic mechanical analysis (DMA) on a DMA 1 device from Mettler Toledo (Zurich, Switzerland). The studies were carried out in air at a heating rate of 10 °C/min in the case of DSC, 2 °C/min, and a frequency of 1 Hz in the case of DMA.

The thermal stability of PE/EVA blends was evaluated using thermogravimetric analysis (TGA) on a TGA 4000 instrument from PerkinElmer (USA) at a heating rate of 5 °C/min in the air environment.

Samples were printed on a Roboze One 3D printer (Bari, Italy) at a nozzle temperature of 200 °C and various desktop temperatures. Printing was carried out in the following modes: degree of filling 100%; orientation of the rasters +45/−45; printing speed 30 mm/s; nozzle diameter 0.4 mm; layer height 0.2 mm.

## 3. Results and Discussion

### 3.1. Rheological Properties

**Figure 1:**
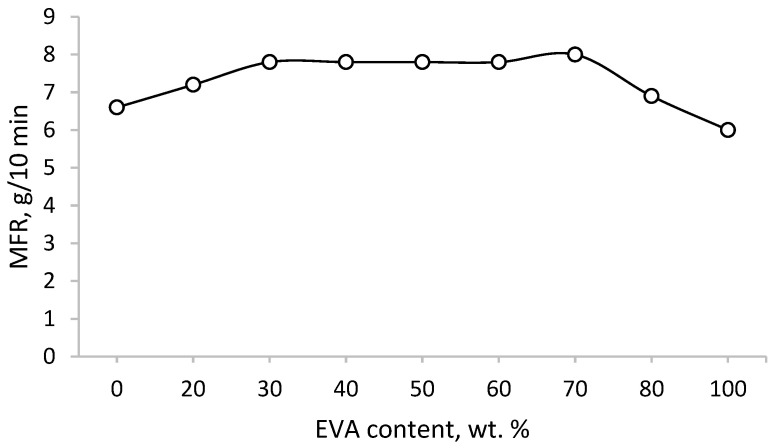
MFR of PE/EVA blends.

A study of blends showed that the introduction of 20% EVA leads to a slight increase in the MFR of PE (from 6.6 to 7.2 g/10 min) (Figure 1). Later, at EVA concentrations from 30 to 70%, the MFR reaches a certain constant value, which decreases only when the EVA content reaches 80%. It is noteworthy that the blends have a higher MFR than the individual components.

Since the MFR corresponds to only one point on the material flow curve and does not provide complete information about the rheological behavior at different shear rates [19], studies were carried out using a capillary rheometer.

**Figure 2:**
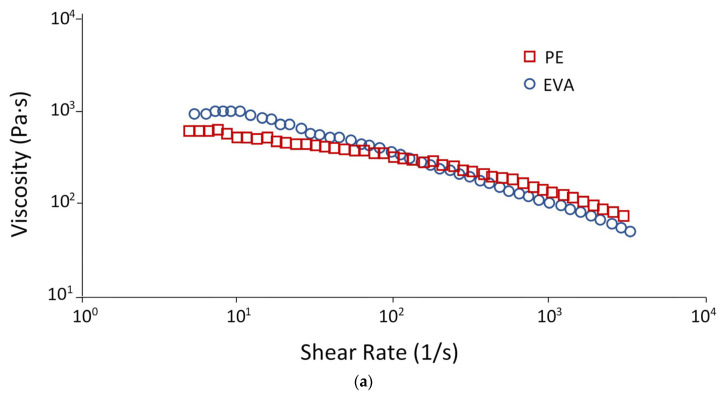

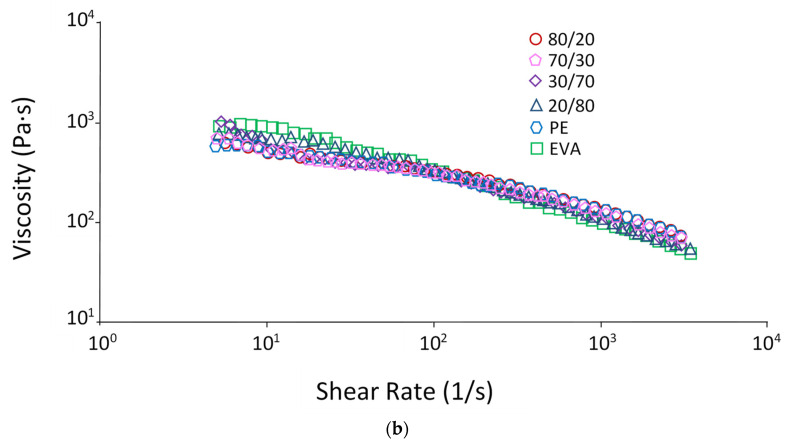
Flow curves: (**a**)—PE and EVA; (**b**)—blends 80/20; 70/30; 30/70; 20/80.

As a rule, polymer melts are pseudoplastic liquids, i.e., their viscosity decreases with increasing shear rate. Figure 2a shows that both PE and EVA at very low shear rates (*γ* < 10 s^−1^) demonstrate Newtonian behavior, as evidenced by the plateau on the flow curves in this region. However, a further increase in the shear rate causes a deviation from the Newtonian flow, i.e., both materials show shear-thinning behavior. To a greater extent, this is typical for EVA: at low shear rates (<100 s^−1^), the viscosity of the EVA melt is higher than the viscosity of PE; at speeds, the viscosity of EVA becomes lower.

The shear-thinning behavior of many polymers can be described by Formula (1), where *η* is the viscosity, *k* is a constant (the consistency coefficient is numerically equal to the stress or viscosity of the fluid at the shear rate), *n* is a power index characterizing the degree of deviation of the flow pattern from the Newtonian law (shear-thinning index). At *n* < 1, the fluid exhibits shear-thinning behavior; at *n* = 0, the fluid is Newtonian; and at *n* > 1, it is dilatant, i.e., viscosity increases with increasing shear rate.

(1) $$\eta =k{\left(\gamma \right)}^{n-1}$$

Figure 2b shows that PE/EVA blends demonstrate averaged viscosity values and an additive character of the flow curves depending on the composition: the higher the EVA content, the greater the shear-thinning effect is observed on the curves. To quantify this property, the values of the exponential index *n* were determined (Table 1), which confirms the observed nature of the change in viscosity. A gradual decrease in n is noticeable with increasing EVA content, which indicates a greater degree of viscosity reduction with increasing shear rate. A more pronounced effect of pseudo-plasticity is a positive factor for materials for 3D printing since an increase in viscosity with a decrease in pressure or its absence (during printing stops) prevents the melt from flowing out of the die, and applying a small pressure, which is typical for 3D printing, contributes to the rapid achievement of required melt rheology.

**Figure 3:**
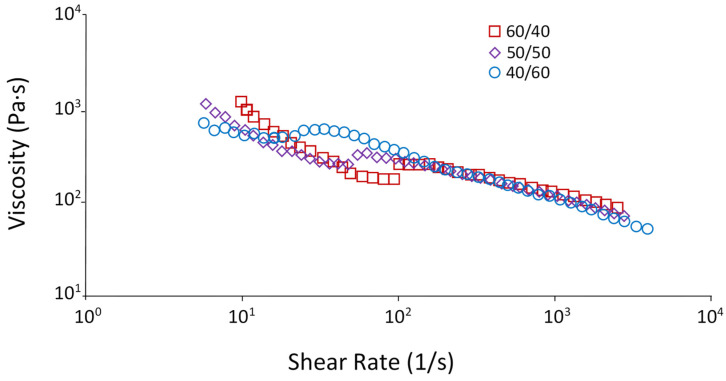
Flow curves of PE/EVA blends.

Noteworthy are the flow curves of blends with compositions 60/40, 50/50, and 40/60 (Figure 3). These materials exhibit anomalous behavior, which manifests itself as a decrease and then an increase in viscosity at certain shear rates and then decrease again. Such a bend in the curve, apparently, is due to phase inversion at the given ratios of the components, i.e., these mixtures are systems with two continuous phases, in contrast to structures with a dispersed phase and a dispersion medium, which are realized at other ratios.

### 3.2. Viscoelastic Properties

**Figure 4:**
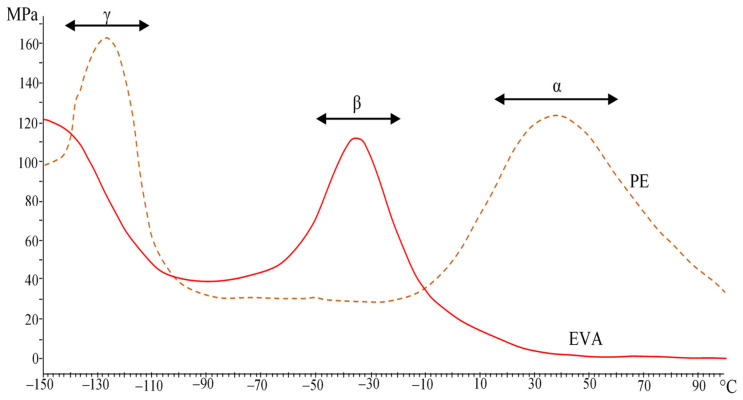
Loss moduli of PE and EVA.

A study of the viscoelastic properties of PE/EVA blends was made. The study by the method of dynamic-mechanical analysis (DMA) was carried out at temperatures from −150 to +100 °C. Figure 4 shows the DMA curves of the initial PE and EVA.

PE, due to its chemical structure and high crystallinity, has three temperature transitions that lie below the melting point, the so-called α-, β-, and γ-transitions [20]. It is generally accepted that the α-transition is associated with the movement of chain links inside the crystalline region [21]. The content of vinyl acetate bulk groups in a random order causes a more amorphous structure of EVA and the absence of an α-transition. The β-transition, which is associated with the movement of chain links located in the interfacial region, manifests itself quite intensively in EVA, in contrast to PE, where only a small shoulder is observed. The origin of the γ-transition is associated with the movement of small segments (several methylene groups) in amorphous regions and can be taken as the glass transition temperature (T_g_). For EVA, this peak lies in the region of lower temperatures.

**Figure 5:**
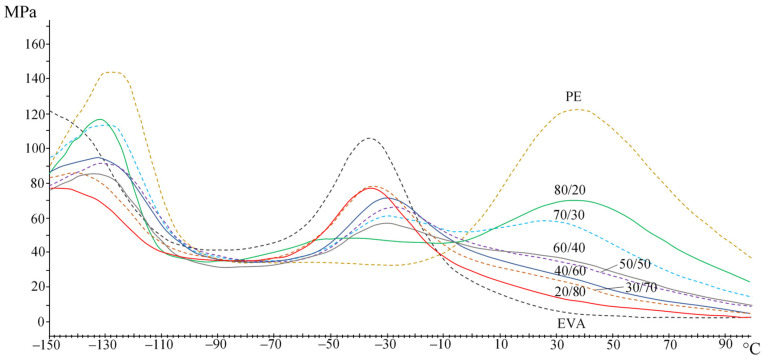
Loss moduli of PE/EVA blends.

The introduction of EVA into PE leads to a gradual decrease in the intensity of the peaks of α- and γ-relaxations and an increase in the β-transition peak (Figure 5). In general, changes occur gradually in accordance with the blend composition. The results are shown in Table 2, where the temperatures corresponding to the peaks of temperature transitions on the curves of the loss modulus were taken to describe the observed effects.

Table 2 shows that with an increase in the EVA content, the temperatures of the α-, β-, and γ-transitions shift to lower temperatures. When the EVA concentration reaches 60%, the α-transition is practically not detected. The decrease in the α-transition temperature is associated with an increase in the mobility of PE chains in the amorphous phase due to EVA plasticization, as evidenced by an increase in the intensity of the peaks of β- and γ-transitions, as well as a decrease in its crystallinity. Single peaks of relaxation transitions indicate the compatibility of these polymers in the amorphous phase.

**Figure 6:**
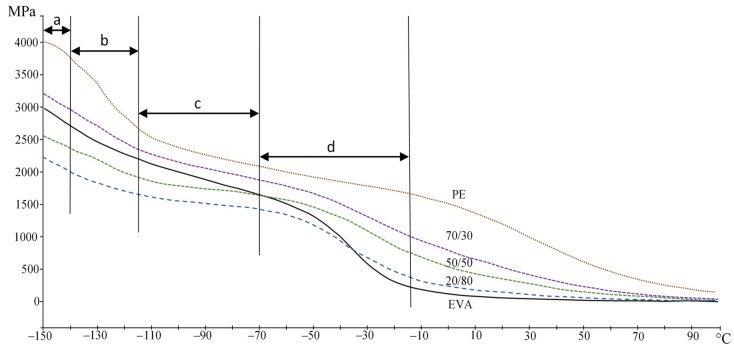
PE/EVA blend storage module.

A decrease in the storage modulus with an increase in the EVA content also indicates a decrease in crystallinity and an increase in free volume (Figure 6). It can be seen (Figure 6) that all compositions have several regions: the region of the glassy state (<−130 °C (a)), the transition region (γ-transition (b)), the region of the highly elastic state (c) and the region of the β-transition (d). For PE, the α-transition is also well observed at higher temperatures.

PE/EVA blends have been studied using DSC. The degree of crystallinity was calculated by Equation (2):

(2) $$X_{c}=\frac{{∆H}_{m}}{{∆H}_{m}^{0}w}\times 100\;\%$$

where ${∆H}_{m}$ is the melting enthalpy of the sample; ${∆H}_{m}^{0}$ is the melting enthalpy of PE in a 100% crystalline state, equal to 293 J/g, which was also used for EVA, in which ethylene segments are also crystallizing units [22,23]; *w* is the mass fraction of the component in the mixture.

The rate of crystallization of PE and EVA in mixtures was also determined, which was estimated from the half-life of crystallization (*t*_½_), i.e., by the time of formation of half of the crystallites during cooling, using the following equations:

(3) $$K=\frac{{∆H}_{cr}}{{2t}_{½}}$$

(4) $$t_{½}=\frac{\left(T_{cr\;onset}-T_{cr\;max}\right)}{V_{cooling}}$$

where $T_{cr\;onset}$ is the initial crystallization temperature; $T_{cr\;max}$ is peak crystallization temperature (at maximum heat release); $V_{cooling}$ is cooling temperature; ${∆H}_{cr}$ is the enthalpy of crystallization.

### 3.3. Thermal Properties

**Figure 7:**
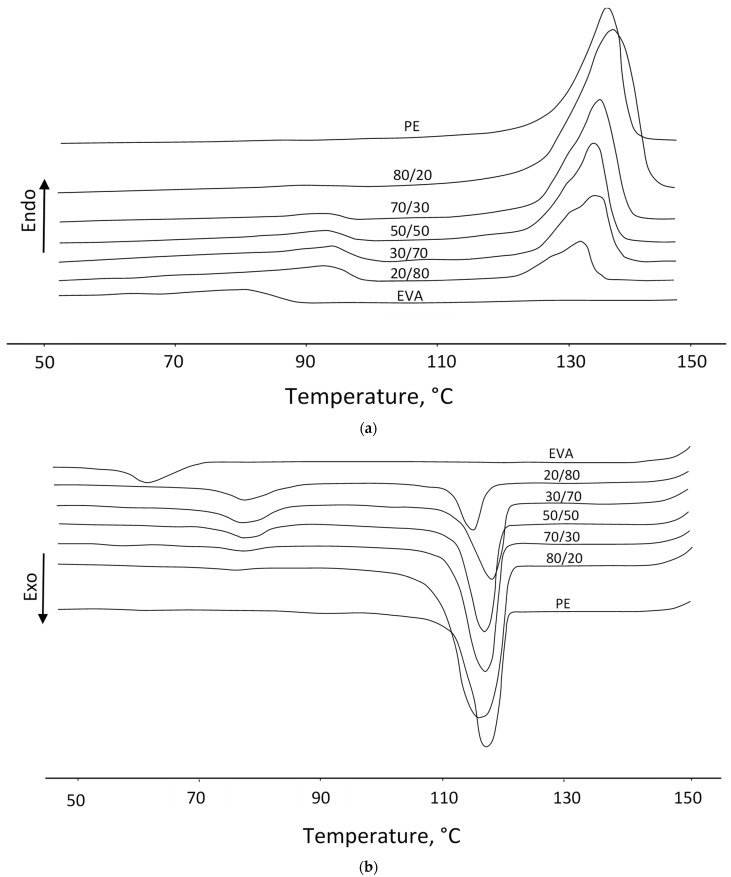
DSC thermograms: melting (**a**) and crystallization (**b**) curves.

The studies performed show that the DSC thermograms of mixtures (Figure 7) show two peaks of melting (second heating) and crystallization (first cooling), which indicates the formation of a two-phase structure of PE and EVA, as reported in a number of works [24,25]. In the case of a low content of EVA (20%), the second endo- and exothermic peak in the temperature range corresponding to the melting and crystallization temperatures of EVA manifests itself rather weakly, but later, the peaks become more intense.

Table 3 shows that when the EVA content is above 20%, the melting temperature (T_m_) of PE crystallites decreases. You can also notice a slight decrease in the degree of crystallinity and crystallization temperature of PE. The data obtained indicate the partial compatibility of PE and EVA in the crystalline phase, i.e., as a result of co-crystallization, less perfect PE crystallites with a lower melting point are formed. In favor of partial compatibility in the crystalline phase, the observed nucleating effect of PE crystallites is also evidenced, which is expressed in an increase in the degree of crystallinity, as well as in the melting and crystallization temperatures of EVA. At the same time, there is a significant decrease in the crystallization rate (K) of PE in the presence of EVA, while in blends enriched with EVA, the crystallization rate of the latter increases (Table 4).

Thus, EVA has a suppressive effect on the processes of PE crystallization. Despite only a slight decrease in the degree of crystallinity and crystallization temperature of PE, its crystallization rate is significantly reduced. Those EVA mainly affect the crystallization kinetics but not the final degree of crystallinity, which was also mentioned in [26]. At the same time, in mixtures enriched with EVA, PE, on the contrary, serves as a crystallization nucleating agent, increasing the melting and crystallization temperatures, as well as the degree of EVA crystallinity. In view of the phase changes revealed using DSC and DMA, it can be assumed that, despite the partial compatibility of PE and EVA in the crystalline phase, the main interaction of the mixed polymers apparently occurs in the amorphous part, which will determine the final properties of the composites as a whole.

**Figure 8:**
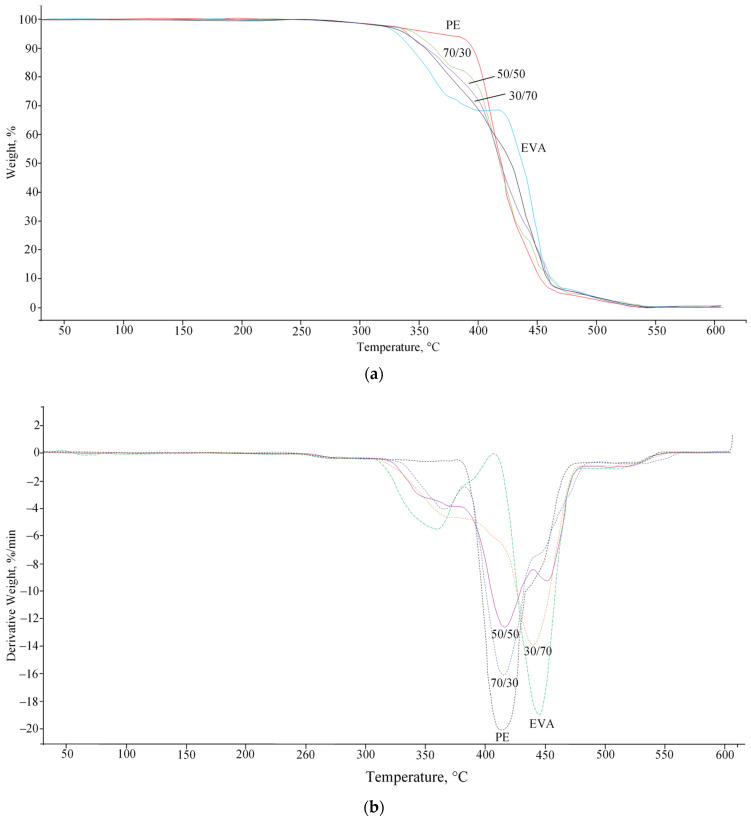
TGA curves (**a**) and derivatives of TGA curves (**b**) of PE/EVA blends.

Thermogravimetric analysis (TGA) showed that PE has a higher degradation start temperature than EVA (Figure 8a). The introduction of the latter leads to a gradual decrease in the thermal stability of mixtures (Table 5). From the derivatives of the TGA curves (Figure 8b), it can be seen that EVA decomposes in two stages: at the first stage (from 310 to 370 °C), the process of deacetylation occurs, at the second (from 420 to 470 °C)—decomposition of the main chain [26]. As the EVA content in the mixture increases, the intensity of the maximum decomposition rate peak in the low-temperature region gradually increases, and the decomposition peak of the main PE chain moves to the region of higher temperatures. In general, the change in heat resistance occurs in accordance with the composition of blends and their initial properties.

### 3.4. Mechanical Properties

The mechanical properties of PE/EVA blends were investigated. Additive values of mechanical properties were calculated using the formula:

(5) $$A=wM_{1}+(1-w)M_{2}$$

where w is the mass fraction; $M_{1}$ and $M_{2}$ are the mechanical property values for components 1 and 2, respectively.

**Figure 9:**
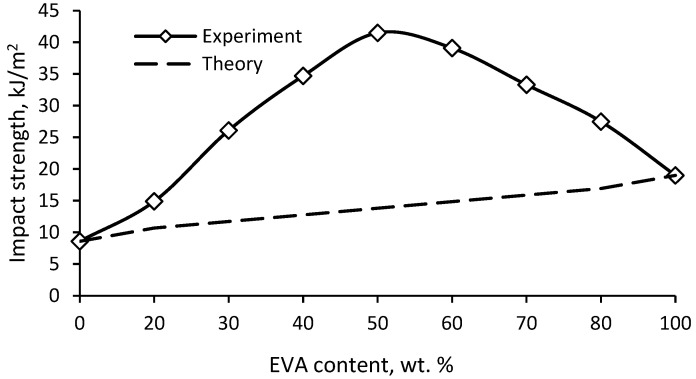
Impact strength (notched) of PE/EVA blends.

The study of impact strength showed (Figure 9) a significant positive deviation of the experimental results from the additive ones (synergism). The additivity rule does not take into account the possible interactions of the components, on the basis of which the deviation of the experimental data from the theoretical values will be due to the mutual influence of the components of the mixture. It is known that for incompatible polymers, the mechanical properties largely depend on the interfacial interaction. However, the properties of compatible polymers determine the degree of compatibility and composition of the composite. Figure 9 shows that the change in impact strength is extreme, with a peak at a ratio of 50/50. Based on the observed synergistic effect, it can be concluded that PE and EVA have fairly good compatibility, which leads to significant plasticization of PE, and at a ratio of 50/50, a morphology with a greater ability to resist crack propagation is formed.

**Figure 10:**
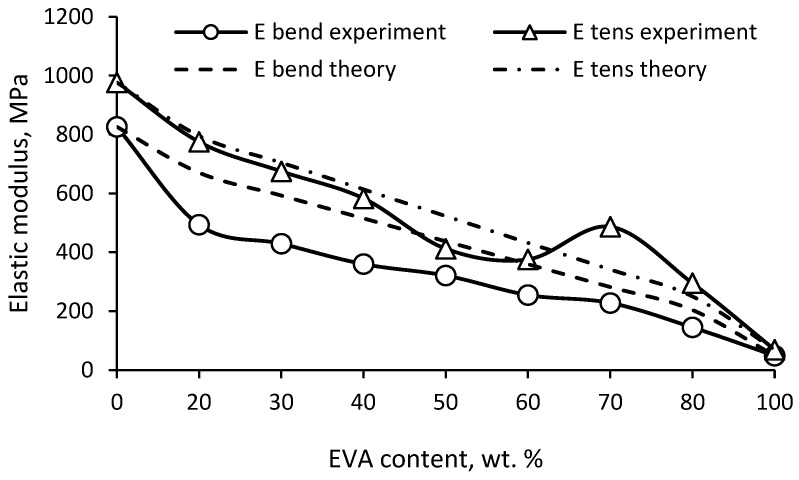
Elastic modulus of PE/EVA blends.

Figure 10 shows the change in bending and tension elastic modulus with the composition of PE/EVA blends. Again, a positive deviation of the experimental values from the additive ones is noticeable, especially for the modulus in bending. Since the degree of crystallinity decreases slightly in mixtures enriched with PE, the observed sharper drop in the flexural modulus in comparison with the expected theoretical values is apparently due to the plasticizing effect of EVA, which consists of an increase in the free volume and mobility of chains in the amorphous part of PE due to the bulk side group of vinyl acetate.

**Figure 11:**
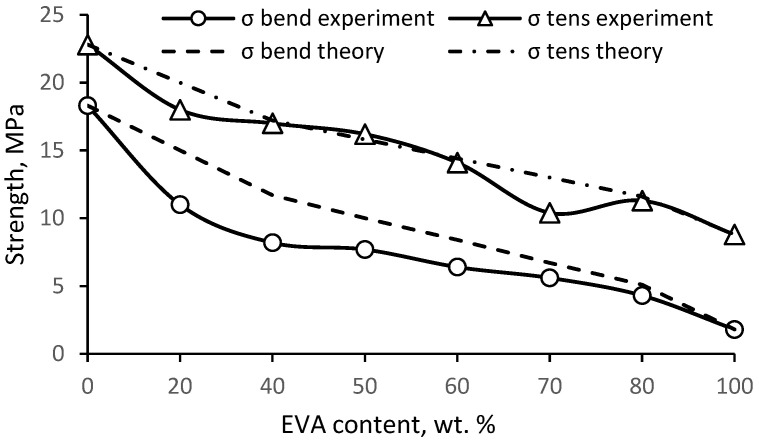
Strength of PE/EVA blends.

Strength, both in bending and in tension, decreases uniformly with increasing EVA content (Figure 11). There is also a slight positive deviation from the additive values, especially in the case of flexural strength, which seems to be due to the same reasons. The tensile strength is in better agreement with the theoretical values.

### 3.5. Filament Making and 3D Printing

Thus, several blend compositions were taken for testing in 3D printing using the FDM method. In the first stage, the manufacturability of materials was studied when obtaining filaments with a certain diameter (1.75 mm). The filaments were cooled using a water bath. Extrusion modes and filament parameters are given in Table 6.

Table 6 shows that the PE filament forms an oval geometry. D1 and D2 correspond to the major and minor axes of the ellipsoid. The larger diameter corresponds to the direction of the laser meter, and the smaller diameter corresponds to the perpendicular direction subject to shrinkage. The given data are the average values of a series of measurements carried out on 3 m of thread in increments of approximately 15 cm (20 measurements were carried out). Filaments with 30 and 50% EVA have a significantly lower ovality (by a factor of 4–5), which is apparently associated with a decrease in the rate of PE crystallization and, accordingly, shrinkage upon cooling. However, a further increase in the EVA content leads to an inverse increase in shrinkage: it can be seen that the filament with the content of 20% PE in EVA (20/80) has the highest shrinkage. As mentioned above, PE has a nucleating effect on EVA; PE crystallites become EVA crystallization nuclei, which leads to an increase in its degree of crystallinity and crystallization rate, as shown in Table 3 and Table 4. Thus, the filament shrinkage data are in good agreement with the crystallization kinetics data.

The FDM printing process places special demands on new materials. There are several problems that can disrupt the printing process: firstly, an inaccurate filament diameter that prevents the melt from being fed; secondly, the bending of the filament during extrusion in the case of low-modulus materials. In this regard, the filament must have balanced physical properties in the solid state, in particular, the compression modulus and rheological properties in the molten state.

**Figure 12:**
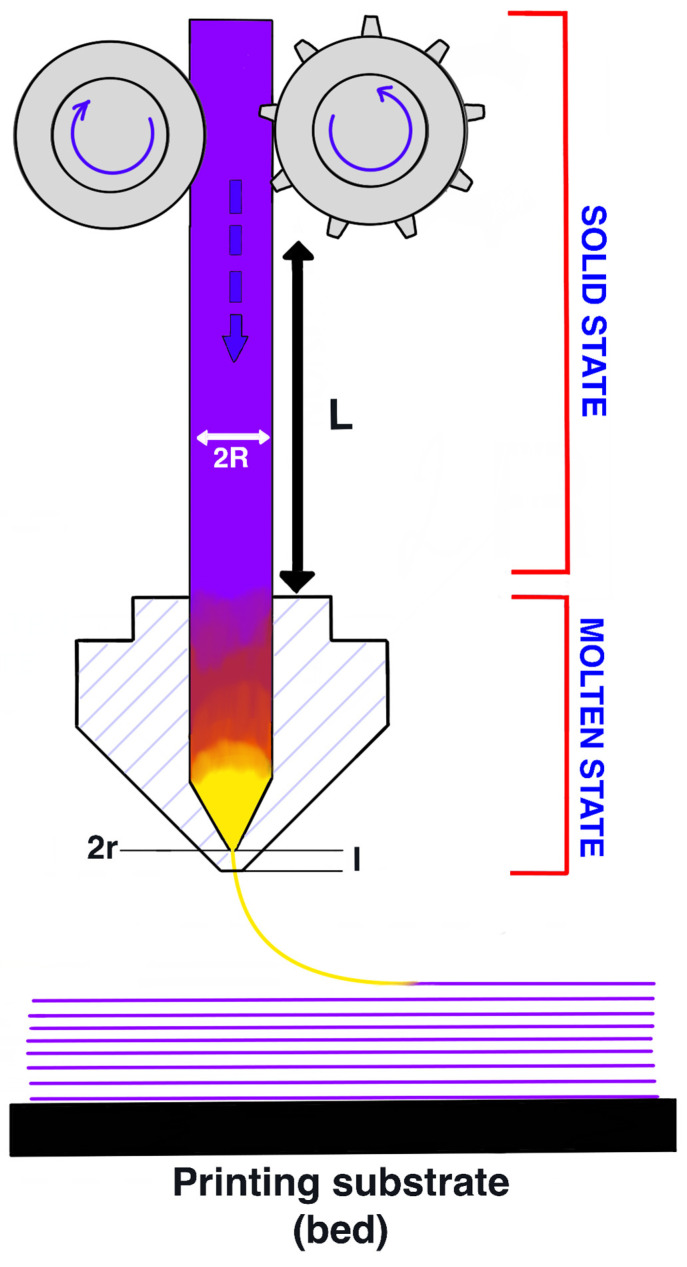
Diagram of the FDM printing process.

The critical stress that a filament can withstand without bending is calculated using the formula:

(6) $$\sigma_{cr}=\frac{K\pi^{2}}{{\left(\frac{L}{R}\right)}^{2}}$$

where K is the modulus of elasticity in compression, *L* is the distance between the rollers and the print head, R is the radius of the filament (Figure 12).

The pressure required to extrude a pseudoplastic fluid at a given shear rate (*γ*) through a die nozzle with radius *r* (0.2 mm) and length *l* (1 mm) can be calculated using the equation:

(7) $$∆P=\frac{8Q\eta_{a}l}{\pi r^{4}}$$

(8) $$Q=\frac{\pi d_{f}^{2}\nu }{4}$$

where Q is the volume flow rate, $d_{f}$ is the filament diameter, ν is the printing speed (30 mm/s).

The shear rates at the wall ($\gamma_{w}$) experienced by the material passing through a die with a round hole can be expressed as follows:

(9) $$\gamma_{w}=\frac{32Q}{\pi d_{n}^{3}}\;\left(\frac{3n+1}{4n}\right)$$

where $d_{n}$ is the die hole diameter (nozzle 0.4 mm), and *n* is the power index of pseudoplastic flow.

For successful printing of the material, the critical filament bending stress ($\sigma_{cr}$ critical buckling stress) must be greater than the pressure created inside the die ($\sigma_{cr}\;$> ∆P). When this condition is met, the necessary volumetric flow during printing is provided [27,28]. Thus, the filament must have the necessary rigidity and bending strength to extrude the viscous melt from the nozzle. In the case of a high melt viscosity and a low elastic modulus of the filament, this condition will not be met, which will lead to filament bending or unstable melt flow.

**Figure 13:**
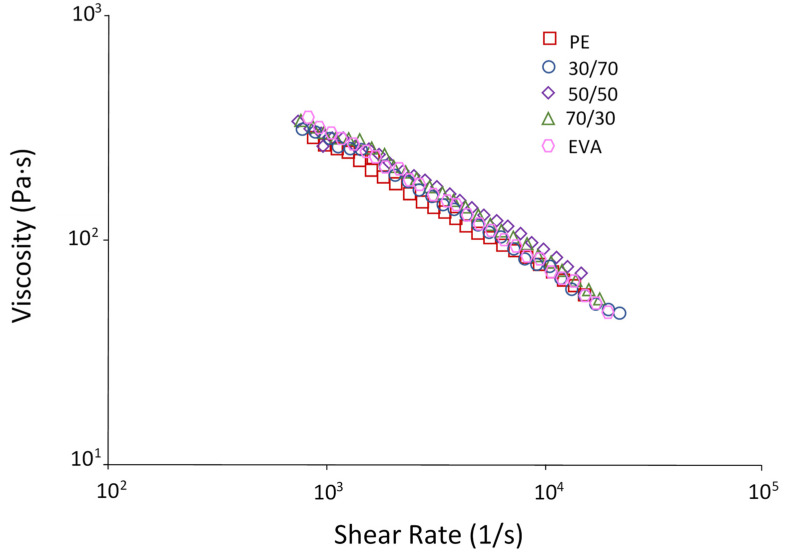
Melt viscosity of PE/EVA blends.

The values of the shear rate ($\gamma_{w}$) calculated using Formula (9) under printing conditions (Table 4) turned out to be significantly higher than those used in rheological studies of blends (Figure 2), where the maximum value was about 3 × 10^3^ s^−1^. In this regard, measurements were carried out at a higher $\gamma_{w}$ (up to 1.5 × 10^4^), which made it possible to reveal the viscosity of the melt under printing conditions. It can be seen from Figure 13 that at high shear rates, the blends have rather close melt viscosities.

The resulting viscosity values were used to calculate the required extrusion pressure. Table 7 shows that all materials have significantly lower values of critical stress $\sigma_{cr}$ than the pressure required to extrude a melt of a given viscosity (∆P). Failure to meet the condition $\sigma_{cr}$ > ∆P means that all filaments must be bent under load during printing.

**Figure 14:**
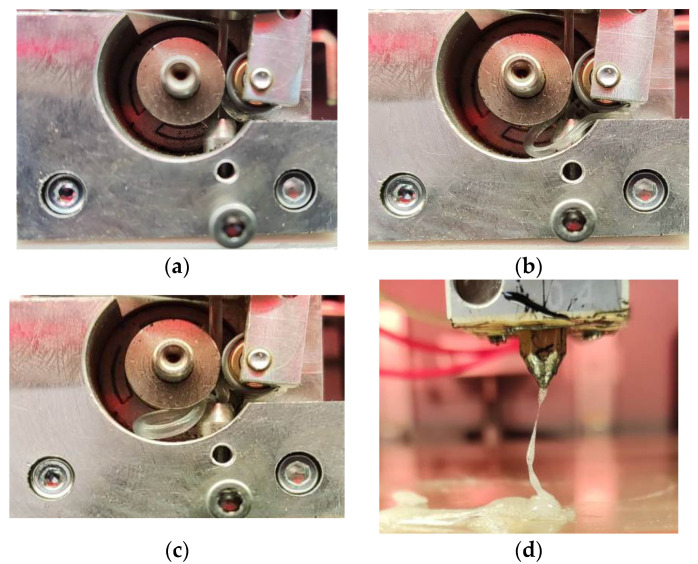
EVA filament extrusion at different nozzle temperatures: (**a**)—200 °C; (**b**)—240 °C; (**c**)—280 °C; (**d**)—300 °C.

However, studies have shown that all materials, with the exception of EVA, extrude well. In the case of the latter, the filament is bent, which makes it impossible to extrude it even when the temperature rises to 300 °C (Figure 14). When the maximum temperature is reached, EVA decomposes (Figure 14d), which corresponds to the temperature of the beginning of its destruction according to TGA data (Figure 8). At the same time, good adhesion of EVA to the bed was noted.

**Figure 15:**
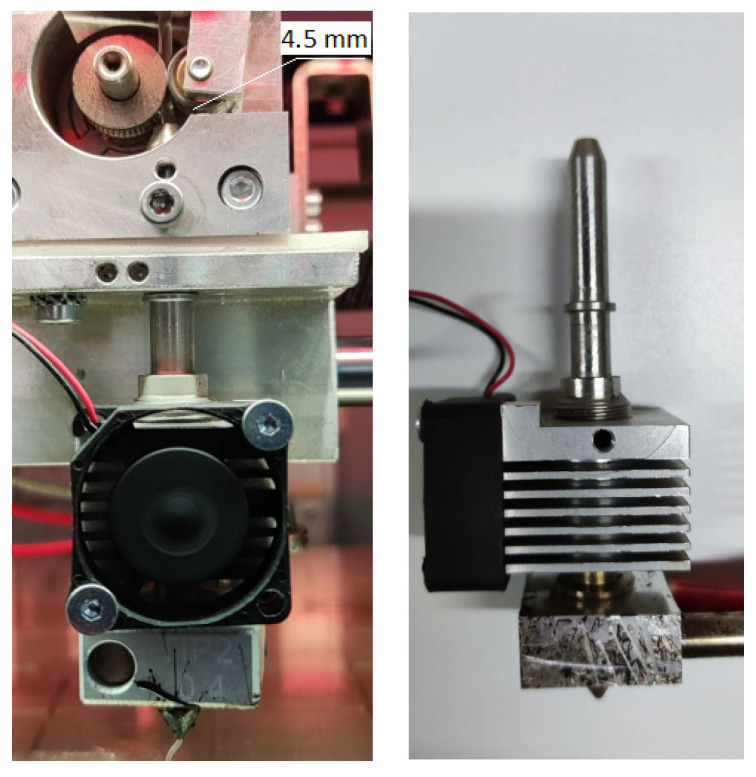
The print head of the Roboze One 3D printer.

The ability of filaments from mixtures to be extruded despite their non-compliance with the condition $\sigma_{cr}$ > ∆P seems to be related to the critical length of the filament. To calculate the critical stress ($\sigma_{cr}$) (Formula (3)), the distance between the rollers and the print head L was used, which was taken equal to 70 mm, which corresponds to the theoretically required value, i.e., the distance between the place of application of the load on the filament and the beginning of the region of its melting. However, in the case of the Roboze One printer, there is an additional metal tube with an inner diameter of 2 mm (Figure 15), in which the filament is held quite tightly and which structurally allows you to avoid bends even if the above condition is not met. Thus, the real space where filament bending can occur is the area between the rollers and the entrance to the metal tube (a distance of about 4.5 mm), which was observed in the case of EVA (Figure 14).

To evaluate and predict this phenomenon, the critical filament length $L_{cr}$ was calculated using the following equation:

(10) $$L_{cr}=\frac{{\pi d}_{f}}{4}\sqrt{\frac{K}{∆P}}$$

where $d_{f}$ is the filament diameter, K is the compression modulus, ∆P is the extrusion pressure.

The data obtained show (Table 7) that for all blends, the value of the critical length significantly exceeds the distance between the rollers and the entrance to the tube of the print head, which ensures the possibility of their extrusion, while the value of $L_{cr}$ for EVA is less than this distance, which is the reason for the bending of the filament from this material.

**Figure 16:**
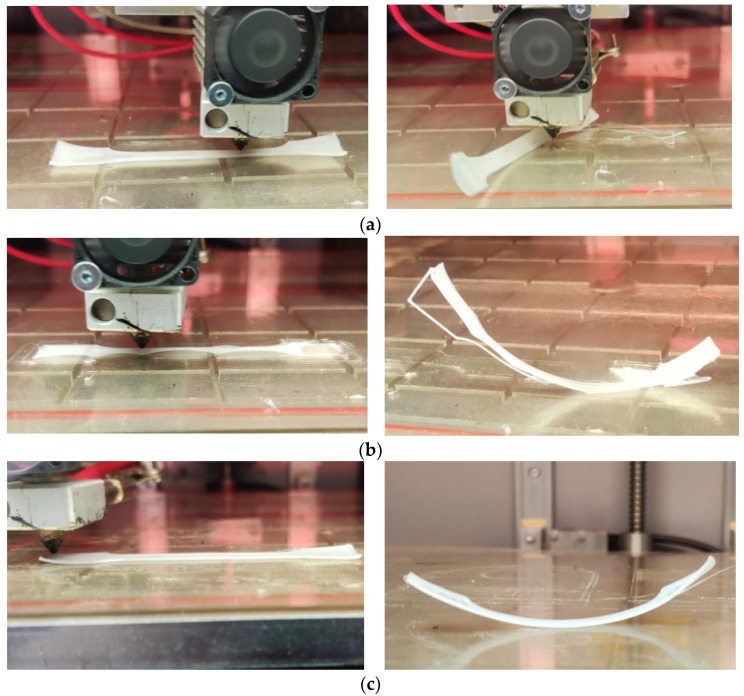
PE printing at a nozzle temperature of 200 °C and a bed temperature of 30 (**a**), 60 (**b**) and 90 (**c**) °C.

Subsequently, the quality and the possibility of printing the resulting mixtures were evaluated. As seen in Figure 16, when printing PE, significant shrinkage and warpage are observed, which leads to the detachment of the edges of the sample from the surface of the bed, followed by its displacement by the extruder from its original location (Figure 16a). With an increase in the bed temperature to 60 °C, warpage occurs to an even greater extent (Figure 16b). Apparently, this temperature provides more favorable conditions for intensive crystallization. A further increase in the bed temperature to 90 °C is also accompanied by a significant change in the sample geometry (Figure 16c).

**Figure 17:**
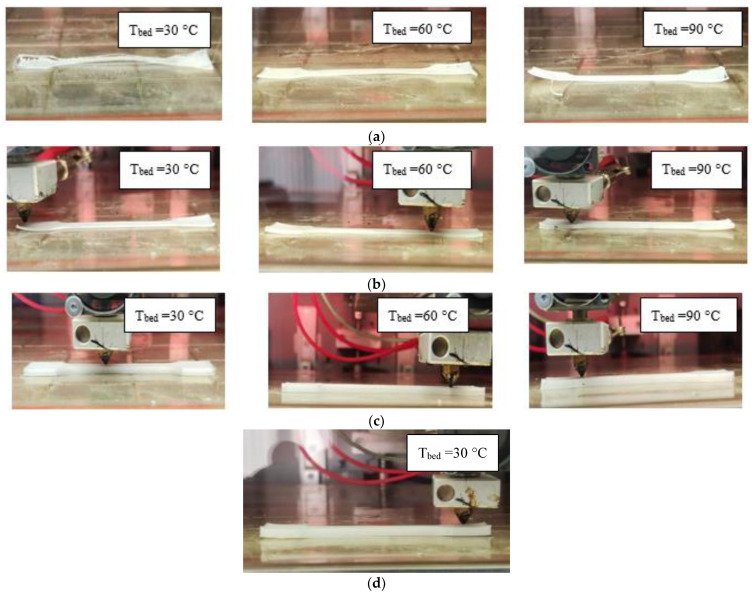
Printing of PE/EVA blends: (**a**)—70/30; (**b**)—50/50; (**c**)—30/70; (**d**)—20/80, at a nozzle temperature of 200 °C and various bed temperatures (T_bed_).

Later, the process of printing PE/EVA blends under similar conditions was studied (Figure 17). In Figure 17a, it can be seen that the 70/30 blend also exhibits poor adhesion to the desktop and significant shrinkage; however, as the EVA content increases, printability increases and warpage decreases.

### 3.6. Warping of 3D Printed Samples

**Figure 18:**
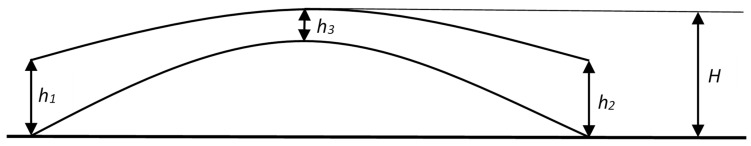
Schematic representation of warping of the dog-bone sample.

For quantitative evaluation, the warping of the samples was characterized by the amount of deviation from the plane, i.e., the maximum vertical displacement of the lower edge of the dog-bone sample (Figure 18), which was calculated by the formula [14]:

(11) $$DP=H-h_{3}-\left(\frac{h_{1}+h_{2}}{2}-h_{3}\right)$$

Table 8 shows that an increase in the EVA content allows an increase in the number of printed layers before stripping and significantly reduces warping by 3–5 times for compositions with a ratio of 70/30 and 50/50. Increasing the desktop temperature has a positive effect on the process of printing blends with EVA, in contrast to the original PE. Apparently, EVA, due to the polar acetate group and low crystallinity, improves adhesion to the desktop. When printing a 30/70 compound, the warpage is reduced to a minimum, allowing the sample to be completed. When using a composite with a ratio of PE and EVA 20/80, it is also possible to complete the printing of samples; however, a reverse increase in warpage is observed, which is consistent with previous data on filament shrinkage and an increase in the crystallization rate and crystallinity of EVA in a blend of this composition.

## 4. Conclusions

Thus, blends of PE and EVA of various compositions have been studied. Both polymers were found to exhibit shear-thinning melt behavior, which is more pronounced for EVA. A DMA study shows that with an increase in the content of EVA, a decrease in the peak of the α transition occurs. At the same time, an increase in the peaks of β and γ transitions is observed, which indicates the amorphization and plasticization of PE. Single peaks of relaxation transitions indicate the compatibility of PE and EVA in the amorphous phase.

The DSC method revealed that PE and EVA form a two-phase structure; however, a decrease in the melting point of PE indicates their partial compatibility in the crystalline phase. It was found that EVA significantly reduces the crystallization rate of PE while the degree of crystallinity decreases slightly. It was also found that PE, on the contrary, has a nucleating effect on EVA, increasing its degree of crystallinity and crystallization rate.

TGA studies have shown that EVA has a significantly lower heat resistance compared to PE, and an increase in its content in blends leads to a gradual decrease in the heat resistance of mixtures as a whole.

It has been found that EVA leads to a significant increase in the impact strength of notched PE. At the same time, the impact strength shows a significant positive deviation from the additive values, which indicates good compatibility of the mixed polymers. EVA also leads to a consistent decrease in the elastic modulus and strength of PE, the values of which also have a slight positive deviation from the calculated values.

The obtained filaments for printing are characterized by a certain ovality due to their shrinkage, which decreases with increasing EVA content and reaches a minimum value at a PE/EVA ratio of 30/70. At the same time, the composition 20/80 shows an inverse increase in ovality, which is associated with the nucleating effect of PE and, as a consequence, an increase in the degree of crystallinity and crystallization rate of EVA.

The calculation of the melt shear rate during printing made it possible to determine the values of the melt viscosity, which were used to calculate the pressure created in the printer’s extruder (∆P) at a given volumetric flow. The critical bending stresses of the filaments ($\sigma_{cr}$) are also calculated. It has been found that the ability of filaments to extrude despite the non-compliance with the condition $\sigma_{cr}$ > ∆P is associated with the design of the printer head, which makes it possible to increase the critical length of the filament.

It was revealed that PE has low adhesion to the bed and greatly changes the geometry during cooling. The introduction of EVA reduces the warping of PE and allows you to increase the number of printed layers; however, printing can be completed only when using compositions 30/70 and 20/80. At the same time, in the first case, the warpage reaches its minimum values, and in the case of the 20/80 composition, a reverse increase in the warpage of the sample is observed, which is also associated with a higher crystallinity and crystallization rate of EVA in this blend.

## Figures and Tables

**Table 1:** Dependence of the power index *n* on the blend composition.

PE/EVA Ratio	Power Index n
100/0	0.688
80/20	0.715
70/30	0.686
30/70	0.658
20/80	0.615
0/100	0.585

**Table 2:** Temperature transitions of PE/EVA blends.

PE/EVA Ratio	Temperature Transition, °C		
	γ	β	α
100/0	−130.4	-	36.4
80/20	−132.4	(−26.2)	37.2
70/30	−127.0	−28.9	26.0
60/40	−131.7	−29.9	16.2
50/50	−133.1	−30.7	17.4
40/60	−136.0	−32.6	(15.0)
30/70	−135.4	−33.1	(14.9)
20/80	−137.7	−34.2	-
0/100	−143.0	−36.0	-

**Table 3:** Results of studying PE/EVA blends by DSC.

PE/EVA Ratio	T_m_, °C		T_cr_, °C		*X*, %	
	PE	EVA	PE	EVA	PE	EVA
100/0	135.9	-	117.4	-	63.7	-
80/20	136.6	90.8	116.0	76.4	63.8	1.9
70/30	134.6	92.3	117.1	77.2	60.1	4.5
50/50	134.0	93.1	117.0	77.8	53.2	5.5
30/70	134.2	93.2	118.2	78.0	56.0	5.3
20/80	131.8	92.6	114.9	78.1	56.6	7.0
0/100	-	81.2	-	62.0	-	1.9

**Table 4:** Crystallization parameters of PE/EVA blends.

PE/EVA Ratio	T_cr_ (Max), °C		T_cr_ (Onset), °C		ΔH_cr_, J/g		t_½_, s		K, J/g/s	
	PE	EVA	PE	EVA	PE	EVA	PE	EVA	PE	EVA
100/0	117.4	-	122.5	-	194.3	-	30.6	-	3.2	-
80/20	116.0	76.4	122.5	82.9	157.6	2.3	39.0	39.0	2.0	0.03
70/30	117.1	77.1	122.1	85.8	130.8	7.5	30.0	52.2	2.2	0.07
50/50	117.0	77.8	122.0	87.6	80.5	13.0	30.0	58.8	1.3	0.11
30/70	118.2	78.0	122.0	89.0	53.9	17.2	22.8	66.0	1.2	0.13
20/80	114.9	78.1	121.8	89.2	34.0	22.7	41.4	66.6	0.4	0.17
0/100	-	62.1	-	74.0	-	16.6	-	71.4	-	0.12

**Table 5:** TGA results for PE/EVA blends.

PE/EVA Ratio	T_5%_, °C	T_10%_, °C	T_50%_, °C
100/0	368.8	395.7	418.9
80/20	351.5	364.0	432.4
70/30	346.9	363.0	417.9
50/50	341.2	357.5	418.5
30/70	339.6	355.5	429.1
20/80	340.9	353.0	425.1
0/100	331.6	343.2	437.3

**Table 6:** Filament production modes and their diameters.

PE/EVA Ratio	Extrusion Temperature, °C	Diameter of Filaments, mm		Ovality, mm
		D1	D2	(D1 − D2)
100/0	180	1.93 ± 0.07	1.69 ± 0.07	0.24
70/30	175	1.72 ± 0.05	1.66 ± 0.03	0.06
50/50	175	1.73 ± 0.05	1.68 ± 0.05	0.05
30/70	175	1.71 ± 0.04	1.61 ± 0.05	0.10
20/80	175	1.91 ± 0.05	1.70 ± 0.05	0.21
0/100	165	1.75 ± 0.07	1.66 ± 0.07	0.09

**Table 7:** Technological properties of PE/EVA blends.

PE/EVA Ratio	$\gamma_{w}$, s^−1^	Power Law Index n	η, Pa·s	K, MPa	$\sigma_{cr}$, MPa	∆P, MPa	*Lcr*, mm
100/0	1.3 × 10^4^	0.438	60	665.0	1.09	6.2	14.2
80/20	1.3 × 10^4^	0.442	60	539.0	0.83	6.2	12.8
70/30	1.3 × 10^4^	0.433	62	527.3	0.76	6.4	12.5
50/50	1.3 × 10^4^	0.514	75	445.5	0.65	7.7	10.4
30/70	1.3 × 10^4^	0.452	58	259.5	0.36	6.0	9.0
20/80	1.3 × 10^4^	0.454	60	170.0	0.28	6.2	7.2
0/100	1.4 × 10^4^	0.408	60	51.5	0.07	6.2	3.8

**Table 8:** Modes and results of printing PE/EVA filaments.

PE/EVA Ratio	Bed Temperature, °C	Extrusion Temperature, °C	Filament Buckling	Printability	Number of Layers before Separation	Warping (DP), mm
100/0	30	200	No	No	5	12.5
	60		No	No	2	16.8
	90		No	No	2	14.7
70/30	30	200	No	No	1	3.2
	60			No	2	3.4
	90			No	3	4.3
50/50	30	200	No	No	2	4.9
	60			No	4	3.1
	90			Poor	8	3.0
30/70	30	200	No	Yes	No separation	0.34
	60			Yes	No separation	0.25
	90			Yes	No separation	0.29
20/80	30	200	No	Yes	No separation	2.2
0/100	30	200	Yes	-	-	-
		240	Yes	-	-	-
		260	Yes	-	-	-
		280	Yes	-	-	-
		300	Yes	-	-	-

## Data Availability

The data presented in this study are available on request from the corresponding author.
